# Responsive Janus droplets as modular sensory layers for the optical detection of bacteria

**DOI:** 10.1007/s00216-023-04838-w

**Published:** 2023-07-14

**Authors:** Lukas Zeininger

**Affiliations:** Department of Colloid Chemistry, Max Planck Institute of Colloids and Interfaces, Am Muehlenberg 1, 14476 Potsdam, Germany

**Keywords:** Biosensors, Foodborne pathogens, Responsive materials, Emulsions, Stimuli-responsive surfactants, Signal transduction

## Abstract

The field of biosensor development is fueled by innovations in new functional transduction materials and technologies. Material innovations promise to extend current sensor hardware limitations, reduce analysis costs, and ensure broad application of sensor methods. Optical sensors are particularly attractive because they enable sensitive and noninvasive analyte detection in near real-time. Optical transducers convert physical, chemical, or biological events into detectable changes in fluorescence, refractive index, or spectroscopic shifts. Thus, in addition to sophisticated biochemical selector designs, smart transducers can improve signal transmission and amplification, thereby greatly facilitating the practical applicability of biosensors, which, to date, is often hampered by complications such as difficult replication of reproducible selector-analyte interactions within a uniform and consistent sensing area. In this context, stimuli-responsive and optically active Janus emulsions, which are dispersions of kinetically stabilized biphasic fluid droplets, have emerged as a novel triggerable material platform that provides as a versatile and cost-effective alternative for the generation of reproducible, highly sensitive, and modular optical sensing layers. The intrinsic and unprecedented chemical-morphological-optical coupling inside Janus droplets has facilitated optical signal transduction and amplification in various chemo- and biosensor paradigms, which include examples for the rapid and cost-effective detection of major foodborne pathogens. These initial demonstrations resulted in detection limits that rival the capabilities of current commercial platforms. This trend article aims to present a conceptual summary of these initial efforts and to provide a concise and comprehensive overview of the pivotal kinetic and thermodynamic principles that govern the ability of Janus droplets to sensitively and selectively respond to and interact with bacteria.

**Graphical abstract:**

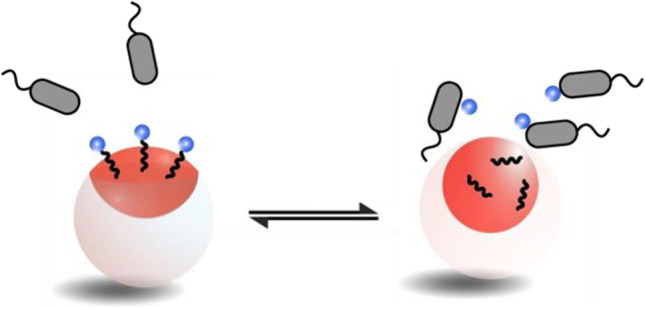

## Introduction

The detection of pathogen-contaminated food is a global public health challenge. [1] Foodborne diseases can cause severe symptoms, including nausea, vomiting, diarrhea, gastroenteritis, and infections that pose a serious risk of illness, particularly for children, the elderly, and immunocompromised individuals. [2] Amongst the various sources of infection, foodborne illnesses are frequently caused by bacteria such as *Salmonella enterica*, *Escherichia coli*, or *Listeria monocytogenes*. [3] To avoid the spread of contaminated food and infections, rapid and reliable early-stage detection and identification of pathogenic organisms is crucial to safeguarding the food supply. [4] In practice, ISO-certified microplate culturing and colony enumeration methods are most commonly employed as the gold standard for detecting foodborne pathogens. [5] The method is simple, has a high success rate, and is highly cost-effective. It requires, however, long time periods of up to 3–7 days to obtain results. [6] During that time, contaminated food is processed, delivered further down the food chain, and reaches supermarkets and consumers before the test outcomes. Regular food recalls and disease outbreaks result from the associated time delay between production and delivery to a safe negative result. Alongside public health concerns, the total economic burden of such pathogen-related food contaminations and infections, which include the direct costs for food recalls, lost revenue, and brand damage, as well as lost work days and health care expenses of infected patients is substantial. [7].

The key to reducing the risk of food poisoning and safeguarding the food supply lies in a reliable early-stage detection of foodborne pathogens. New sensing paradigms are urgently needed that are cost-effective, labor efficient, highly specific, and detect the target analyte within a single working day at a food production facility. New biosensor technologies promise to overcome the limitations of current sensor platforms. [8–13] Elegantly designed and well-known examples include enzyme-linked immunosorbent assays as well as biosensors that are based on DNA amplification, such as polymerase chain reaction (PCR) assays. [14] Other examples include biosensing techniques based on optical signal amplification using nanomaterials, Förster resonance energy transfer–based molecular chemiluminescent probes, or antibody-functionalized optical waveguides with total internal reflection–based imaging modalities. [15–20].

However, the simple fact that referring to your local newspaper from today leaves a high probability of referencing another foodborne pathogen-caused food recall or even disease outbreak reveals that the efficiency and applicability of these techniques are still in need of improvement. Some methods require long bacterial pre-enrichment and incubation steps. Others have significantly improved time to results but are laboratory-based, require costly equipment, or are complicated to perform. In many cases, biochemical identification assays or read-out techniques are simply prohibitively expensive for a rapid on-site testing of food products and are rather designed for medical diagnostics. The biggest hurdles to overcome, particularly in the context of food monitoring, are the overall cost and practicability of the platform to detect a signal against a complex background noise in order to allow for easy on-site screening of samples. [21].

Liquid–liquid transduction schemes, such as those based on responsive Janus emulsions, are appealing because they are simple and cost-effectively to prepare in any environment from inexpensive commercial reagents and the dynamic liquid interfaces facilitate reactions of synthetic bio-selectors and pathogens within their native aqueous environment. The latter, in principle, allows for an emulation of signal transduction and transmission strategies found in nature, where the highest substrate specificities are often achieved not by interactions between a single selector with a specific analyte but in the form of biochemical logic gates in which multiple independent triggering events actuate a specific reaction cascade. An emulation of such signal selection and amplification strategies requires artificial adaptive material systems that can dynamically respond and adapt to multiple molecular recognition–induced chemical events autonomously. Emulsions, dispersions of fluid droplets stabilized by surfactants, inherently represent a thermodynamically out-of-equilibrium material platform. [22] Emulsion droplets are highly dynamic, with molecules constantly being exchanged between the droplets and their environment. In this regard, they closely resemble cell surface environments and therefore offer multiple enticing opportunities for designing biomimetic signal transduction pathways where they can interact with pathogens similar to how pathogens interact with cells.

While one-component droplets are ubiquitous and have found widespread applications in many commercial products, including paint, food, cosmetics, and as central components of many medical, pharmaceutical, and performance products, the introduction of a third orthogonal liquid phase opens the path towards a variety of new multicomponent systems that display multiple separate responsive modalities. [23] More specifically, dispersions of kinetically stabilized biphasic Janus droplets have recently attracted attention for developing new and improved emulsion technologies. [24] Functionalized biphasic Janus emulsions display high surface areas and dynamic interactions with tailored surfactants allow to mimic and emulate biological interactions at the droplet interface. Small chemical changes in the droplet environment trigger microscopic changes in the shape of the droplets, which in turn vary the optical properties of the droplets on the macro scale. [25, 26] This unique chemical-morphological-optical coupling inside Janus emulsions can be exploited to create a variety of dynamic and stimuli-responsive micro-scale optical components and therefore presents an extraordinary untapped potential for the development and implementation of easily deployable transducers and signal amplifiers for liquid biosensing platforms. Initial demonstrations have showcased that Janus emulsion–based modular sensing layers provide the sensitivity to enable rapid and cost-effective foodborne pathogen detection with detection limits that are already competitive with commercial methods.

This trend article summarizes the initial concepts for implementing responsive Janus emulsions as novel transducers and signal amplifiers in liquid biosensing platforms. The article intends to provide as a resource to both newcomer and experienced researchers in the field and to further stimulate cross-disciplinary research activities in the design of new and improved Janus emulsion sensing platforms. Therefore, as part of a conceptual summary, first the underlying chemical design principles for the generation of these types of stimuli-responsive liquid colloids, the associated implications of their dynamic and reconfigurable droplet geometries in the context of emulating biological recognition events and potential signal transduction pathways to visualize such interactions are discussed. These are followed by an overview of the different Janus emulsion–based pathogen sensing strategies reported to date. Finally, potential opportunities and challenges towards improving the sensitivity and specificity while maintaining the simplicity and cost-effectiveness of Janus emulsion–based sensing paradigms are outlined in the ‘Outlook’ of the article.

## Generation of Janus emulsions

**Fig. 1:**
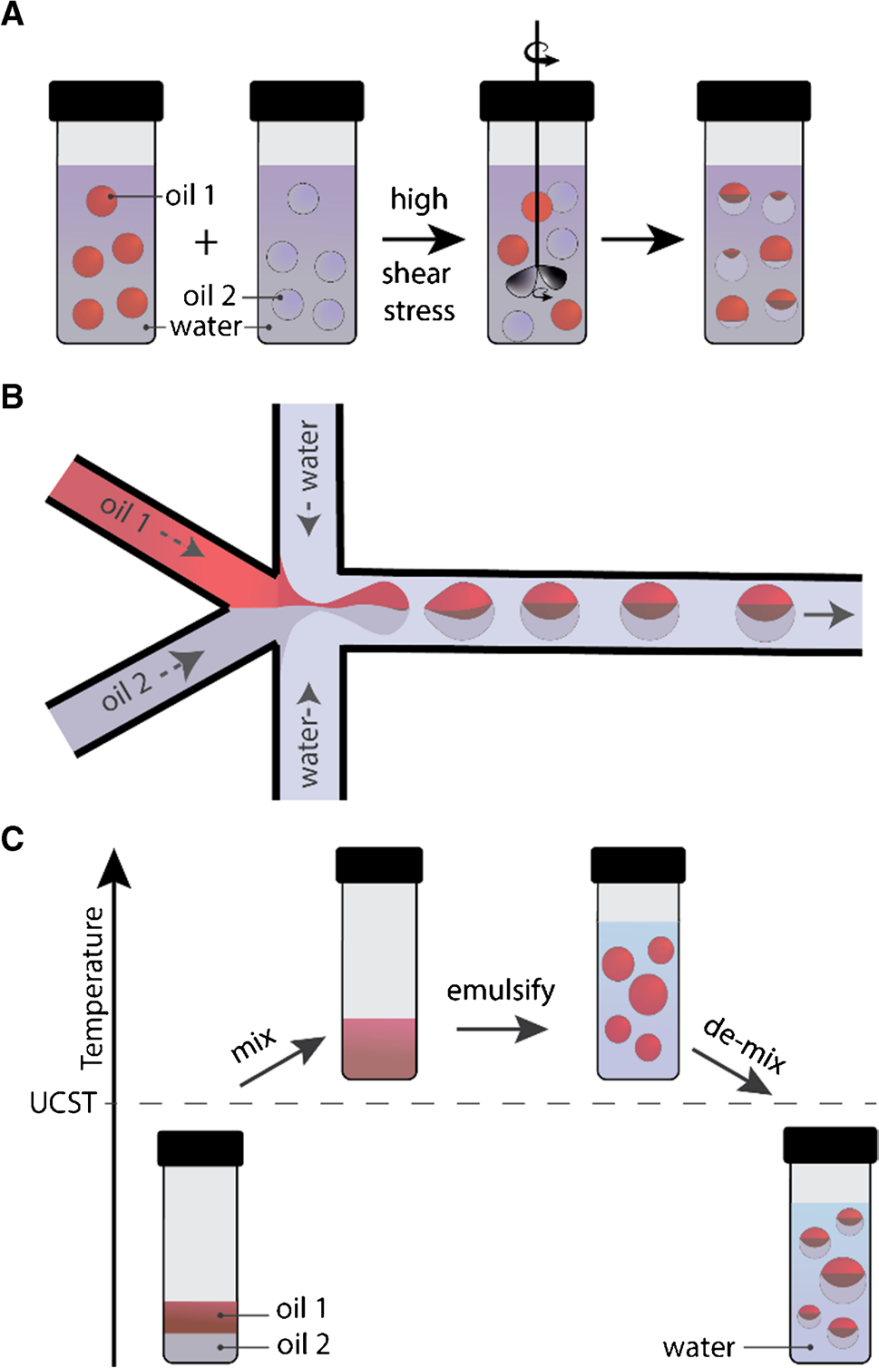
Schematic overview of three general methods to generate Janus emulsion droplets using (**a**) shear stress-induced droplet coalescence, (**b**) sophisticated microfluidic channels, and (**c**) temperature-controlled liquid–liquid phase separation

Per conceptual design, Janus emulsions constitute a ‘smart’ material platform, in which the term ‘smart’ can stand as an acronym for the most compelling characteristics of this material class: self-organized, metastable, artificial, responsive, and triggerable. The droplets are comprised of two immiscible fluids that self-organize into distinct gravitationally aligned compartments creating droplet shapes that are determined exclusively by the force balance of interfacial tensions acting at the individual interfaces. As such, depending on the surfactant composition within the continuous phase, Janus emulsions can be generated in a variety of internal droplet geometries, and small changes in the equilibrium of interfacial tensions evoked by changes in the surfactant type, concentration, or effectiveness allow the droplet geometries to be controllably altered before or after emulsification. A broad compositional flexibility in the selection of the constituent fluids and surfactants as well as a selective partitioning of active components into the individual phases of the droplets enable the generation of a large variety of functional Janus droplets with intricate, switchable, and chemically, physically, electrically, magnetically, or photochemically sensitive structures. These fundamentally new forms of thermodynamically out-of-equilibrium liquid colloids are often simple to prepare from inexpensive commercial reagents in a variety of compositions, geometries, sizes, and size dispersities using different methods that range from large-scale, less precise techniques yielding polydisperse emulsion droplets to more precise but small-volume approaches generating uniformly sized and shaped emulsions droplets. From a fundamental perspective, many reported techniques are based on three underlying principles (Fig. 1): Firstly, as first shown by Torza and Mason, [27] Janus emulsions can be obtained by triggered coalescence of two immiscible liquid droplets suspended in a third immiscible fluid. Coalescence is a physical process in which sufficiently strong mechanical forces are required to overcome droplet–droplet repulsion, which causes two droplets to fuse. [28–31] Secondly, Janus emulsion droplets of highly precise compositions and geometries can be produced by controlled emulsification of multiple liquid phases inside highly sophisticated tapered microfluidic channels. Major advancements in this field stem from Weitz and coworkers, who demonstrated that microfluidic technologies offer excellent control over the flows of multiple fluids to produce various complex emulsions with controllable sizes, shapes, and compartments. [32, 33] Thirdly, using a thermal phase separation approach, Swager and coworkers reported a thermal phase separation method for the bulk generation of Janus emulsions via the emulsification of two liquids above or below their upper or lower critical solution temperature. [34] Returning to room temperature after emulsification results in phase-separation of the two dispersed fluids and consequently, the generation of structured emulsion droplets in highly uniform internal morphologies across a sample. While the phase separation of fluids is thermodynamically controlled, the droplet shape can be governed by the surfactant composition in the continuous phase. Kinetic control over the stability of the individual interfaces allows to controllably alter droplet morphologies before and after droplet generation. The technique applies to many emulsification techniques and therefore represents a paradigm shift for the large-scale generation of a wide range of triggerable and stimuli-responsive Janus droplets and particles. [35–38].

## Janus droplet morphology

**Fig. 2:**
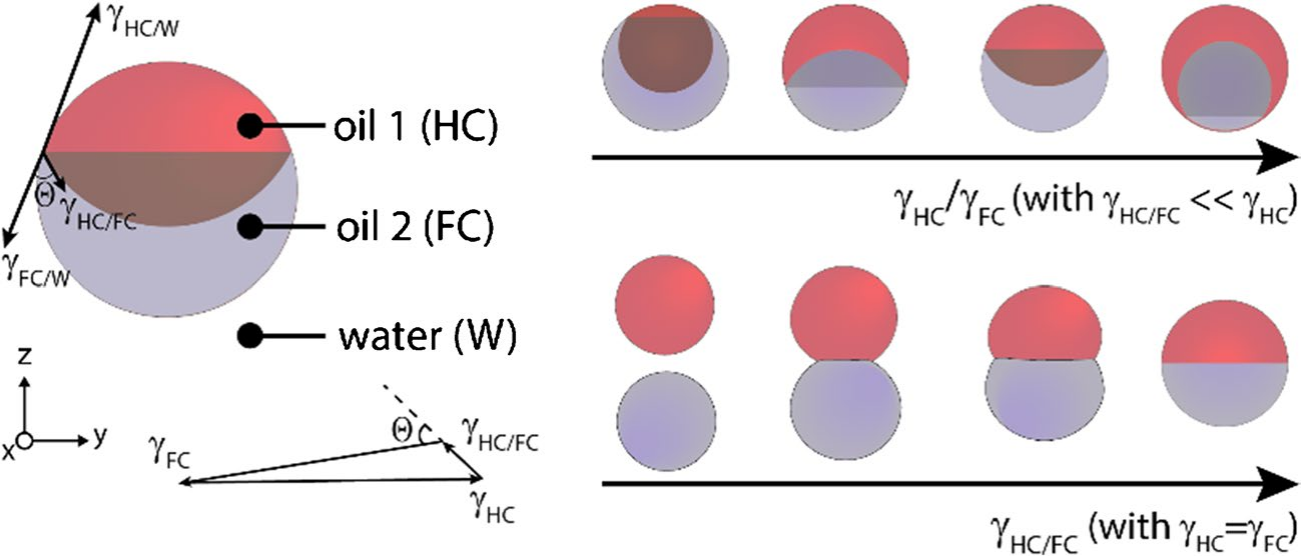
Morphology of Janus emulsion droplets. Sketch outlining the force equilibrium of interfacial tensions acting at the various interfaces that exclusively determines the droplet shape according to the corresponding Neumann’s triangle. These physical relationships set the boundary conditions for the morphological tunability of close-to-spherical Janus droplets as well as they determine the equilibrium shapes of non-spherical droplets

The geometry of Janus emulsion droplets is mutually influenced by the type of dispersed fluids, the volume ratio, and their density. The internal Janus droplet morphology is determined by the force-balance of interfacial tensions acting between the constituent phases as expressed by the Neumann triangle. [39] The physical relationships reveal that if the intrinsic interfacial tension between the constituent solvents of a Janus droplet is large, droplets seek to minimize the interfacial area and the overall shape deviates from a spherical to more ‘snowman’-shaped droplets or droplets become unstable and split up into two separate droplets. In turn, when the interfacial tension between the two internal phases is significantly lower than the interfacial tensions between the continuous and constituent phases, the droplet shape is close to spherical. Examples of the latter include oil-in-water Janus emulsions, i.e., droplets comprising phase separated mixtures of two oils, including hydrocarbons, fluorocarbons, vegetable, mineral, and silicone oils, or liquid crystals dispersed inside an aqueous continuous phase, or, vice versa, water-in-oil droplets with an aqueous multiphase system as the dispersed phase. [38, 40–42] These systems respond to marginal variations in the balance of interfacial tensions at the external interfaces, which creates droplet morphologies that can be dynamically altered also after emulsification. [43] Droplet shapes can be tuned between encapsulated double emulsion and various Janus emulsion topologies, and variations in the external interfacial tensions, e.g., triggered by altering surfactant concentrations or surfactant effectiveness, cause the internal interface of Janus emulsions to be either concave or convex, whereas the overall droplet shape remains spherical. [44] The contact angle at the triple phase contact line determined by lateral imaging of the droplets can be used as a parameter to quantitatively describe particular Janus configurations (Fig. 2). Droplet contact angles range from 0° for an encapsulated double emulsion to 180° for the inverted encapsulated state, with a contact angle of 90° ascribed to the ‘perfect’ Janus state in which the two external interfacial tensions are equal, resulting in a droplet made up of two perfect hemispheres.

## Chemical actuation of changes in Janus droplet morphology

**Fig. 3:**
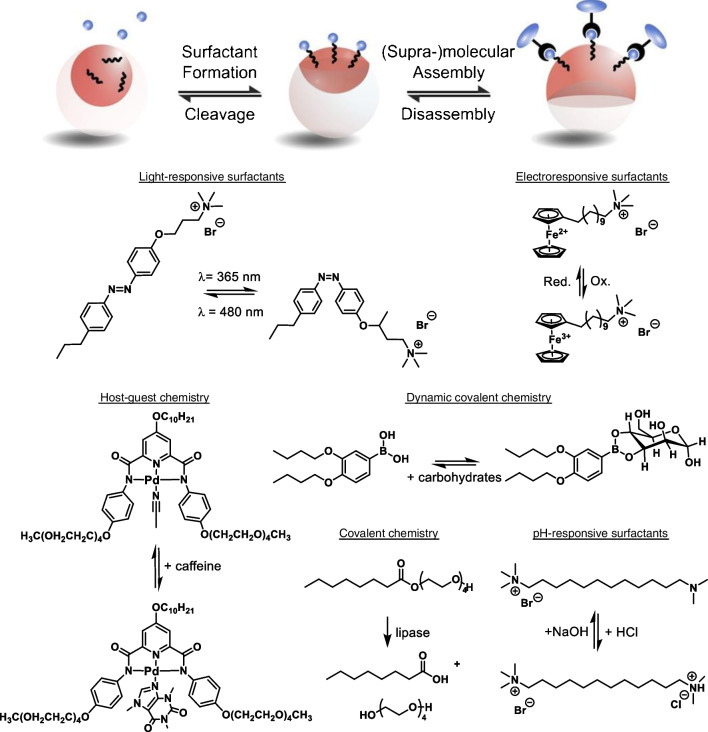
Chemical reactions of analytes with stimuli-responsive surfactants transduce into variations of the internal Janus emulsion morphology. Changes in the surfactant effectiveness can be evoked by cleavage of an existing surfactant, formation of a new or stronger surfactant, as well as via supramolecular, dynamic covalent, or covalent interactions of surfactants with an analyte

Typically, variations in surfactant effectiveness and thus variations of droplet interfacial tensions result in qualitative changes of an emulsion, such as droplet flocculation or coalescence. [45] Leveraging the immediate response of the Janus droplets to dynamic variations in surfactant effectiveness, an in situ monitoring and quantification of changes in surfactant effectiveness in response to external triggers can be realized. The ability to chemically actuate variations in Janus droplet morphology entailed the development of a series of chemically responsive, dynamically reconfigurable emulsion platforms with internal morphologies capable of reporting the presence of specific analytes. In general, these strategies employ an inert surfactant that is required to maintain the stability of the Janus droplets during stimulation in combination with a stimuli-responsive surfactant that is sensitive to a particular analyte. The latter act as chemo-selectors; i.e., they change their effectiveness in response to recognizing a specific chemical entity. Targeted assembly of these responsive surfactants at selective interfaces of the Janus emulsions creates active elements capable of translating chemical recognition events into effective sensory read-out schemes (Fig. 3). A particular advantage of the dynamic hydrophobic-hydrophilic liquid interfaces of the Janus emulsions for biosensor design is that they overcome solubility limitations in chemical selector design as they facilitate reactions between molecular, synthesized sensors that are often associated with limited utility in the native aqueous environment of many target analytes.

The design of amphiphilic surface-active components that allow sensitization of the balance of interfacial tensions of Janus emulsions via supramolecular recognition events is particularly attractive because it offers a multitude of opportunities for the design of Janus emulsion sensing platforms. Supramolecular receptors can be specifically synthesized and compartmentalized into the organic phases of the emulsion droplets. Recognition of water-soluble analytes then changes the surfactant effectiveness, for instance via changing the surfactants’ hydrophilic-lipophilic balance, inducing changes in the respective oil–water interfacial tension. [46] Following these principles, caffeine-sensitive surfactants have been designed based on amphiphilic palladium pincer complexes. [47] The palladium pincer surfactants exhibit a hydrophobic binding pocket for the complexation of *N*-heterocyclic alkaloids. Caffeine binding decreased the surfactant’s hydrophilic-lipophilic balance and consequently resulted in an increase in the respective interfacial tension at the oil–water interface of the Janus emulsion. This method’s utility was illustrated by simply monitoring changes in droplet morphologies as a novel tool for quantifying caffeine levels in sample solutions. [47] Next to the design of artificial receptors, supramolecular functionalized Janus emulsions can also probe biochemical recognition events. Non-covalent interactions are central to many biological processes, and in nature particularly complex networks of protein–protein and protein-carbohydrates play a pivotal role. Zhang et al. demonstrated that stabilizing the droplet interface with carbohydrates allows for interaction with sugar-binding lectins. [48] Janus emulsions were stabilized using a mannose-based surfactant that selectively assembled to one hemisphere of Janus droplets. The addition of concanavalin A, a mannose-binding protein with four mannose-binding subunits, then resulted in multivalent binding to the immobilized sugars at the droplet surface, thereby causing agglutination of the Janus emulsions.

Compared to supramolecular interactions, analyte-induced covalent bond cleavages result in pronounced changes in surfactant efficiency and thus more significant morphology changes can be evoked. The concept was first demonstrated using a pH-sensitive sodium 2,2-bis(hexyloxy)propyl sulfate surfactant. [34] Acid-induced degradation of the surfactant resulted in a complete inversion of droplet morphology from an encapsulated to the inverted encapsulated state. Next to pH-sensitive surfactants, covalent cleavage of an active surfactant was further used to measure and quantify enzyme activity. [49, 50] Molecular cleavage of a surfactant into its hydrophilic and hydrophobic parts or uncaging and release of a surfactant from an enzyme cleavable complex enabled real-time monitoring and quantification of the enzyme activities of amylase, trypsin, lipase, and sulfatase. Covalently triggerable surfactants were further designed to respond to specific nucleophilic stimuli. [51] Specifically, triggerable Michael acceptor functionalities were used as head groups in the surfactant molecules that assemble at the interface of emulsions, and nucleophile-induced modification of the surfactants altered their ability to kinetically stabilize the emulsions.

The advantages of both reversible supramolecular concepts and pronounced droplet responses observed for covalent droplet surface modifications can be leveraged and combined in the development of dynamic covalent interfacial functionalization paradigms. The modularity of the dynamic covalent surfactant manipulation approach was tested using reversible reactions between surface-immobilized boronic acid surfactants and carbohydrates present within the continuous aqueous phase. [26] Boronic acids form reversible cyclic covalent complexes with *cis*-diols. Traditionally, the utility of molecular boronic acid sensors is limited due to the poor water solubility of synthetic boronic acid receptors. [52] In this context, Janus emulsions provide a versatile platform to facilitate reactions between synthetic boronic acid receptors and carbohydrates, yielding sensitive detection schemes for detecting mono- and polysaccharides with up to nano-molar sensitivity. The same reversible binding principles could be extended towards immobilizing glycosylated biomacromolecules, such as for an oriented, side-selective post-functionalization of Janus droplets with antibodies. IgG antibodies present N-glycans as part of their Fc-region, and thus, active antibody binding sites remain available for antigen binding after immobilization. [26, 41].

In an alternative reversible covalent functionalization scheme, Zentner et al. harnessed the dynamic covalent nature of imine bonds. [53] Spontaneous imine-bond formation at the interface of Janus emulsions between surface-active compartmentalized aldehyde reagents and amines within the continuous phase resulted in an in situ formation of stable imine surfactants that provide sufficient stability to Janus emulsions, without the need for additional emulsifiers. The dynamic covalent nature of the imine bond allowed to controllably influence its chemical binding equilibrium, which proved useful in the context of droplet post-functionalization schemes, where Janus emulsion networks could be controllably demulsified on amine-functionalized solid interfaces and Janus droplets could be programmed to respond to amine-containing biomolecular targets. [53, 54].

Next to stimuli-responsive selector surfactants that are specifically designed to interact with a particular analyte, the effectiveness of commercial emulsifiers themselves also depends significantly on the continuous phase chemical environment. Consequently, droplet morphological changes can be observed and monitored in response to minor variations in electrolyte concentrations or pH within the continuous phase. [55–57].

The outlined initial reports on covalent and noncovalent, reversible and irreversible droplet post-functionalization paradigms demonstrate the versatility of actuating dynamic Janus emulsion responses and provide as a basis for a continued merger of complementary synthetic organic chemistry, supramolecular, and catalytic concepts at the dynamic liquid interfaces of Janus emulsions for the targeted design of other versatile responsive Janus emulsion building blocks that exhibit programmable responses to a wide range of chemical stimuli. In the context of developing new, sensitive, and effective chemosensors based on Janus emulsions, a reliable transduction of the unique chemical-morphological coupling inside Janus emulsions into a readable and ideally quantifiable physical signal output is key. In general, chemo-and biosensors typically comprise two main components: [1] a recognition element that selectively binds to a target analyte (selector) and [2] an efficient transduction mechanism that translates the chemical binding events into a quantifiable and, ideally, real-time output signal. Therefore, an efficient mechanism that translates the chemical-morphological coupling into a readable physical signal output is necessary that goes beyond simple microscopic analysis of dynamic droplet responses. Janus emulsions are rich in optical properties rendering them an inexpensive and broadly programmable optical transducer, and initial examples take advantage of these optical features.

## Refractory optical properties of Janus emulsions

**Fig. 4:**
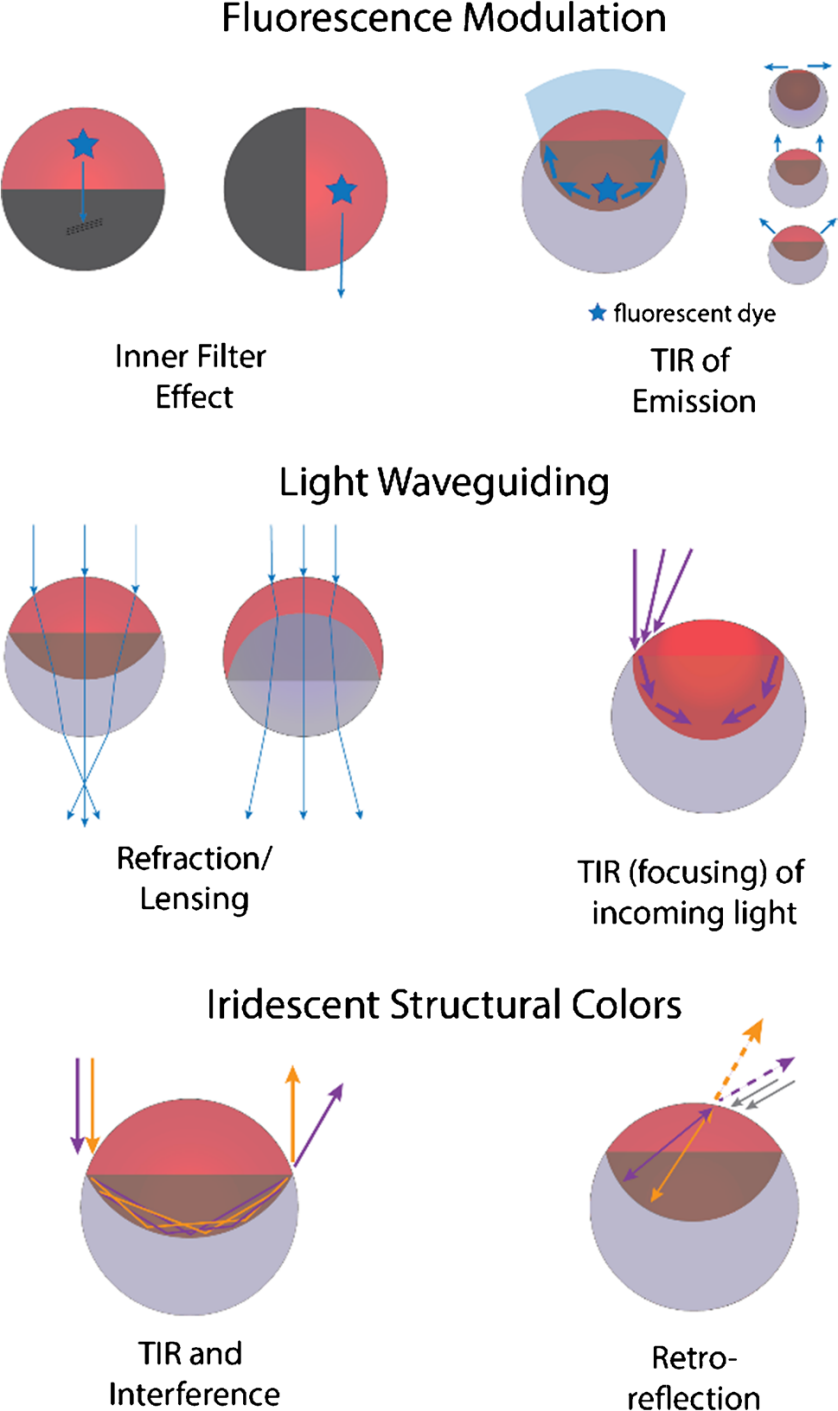
Schematic representation of six examples of the various ways to manipulate the pathway of light to create droplet morphology-dependent optical signal outputs. Figure sketches adapted from references [25, 26, 58–61]

The refractive index contrast between the constituent liquid phases of a Janus emulsion produces reflectivity and optical confinement, which allows modulation of the optical characteristics and to manipulate the pathway of light passing through these microscale elements (Fig. 4). Gravity-induced alignment of the constituent droplet phases underpins the resulting macroscopic optical features. Chemical reactions in the vicinity of a Janus droplet can cause microscale changes in the droplet geometry, which then transduce into visible macro-scale optical effects. Janus droplets have been demonstrated to display versatile refractive, reflective, and light-emitting optical effects that generate unique structural colors, iridescence, light focusing or diverging modes that enable monitoring of chemical recognition events. The refractory optical properties of Janus emulsions can be adjusted by modulating the droplet composition, including the constituent phases’ type, composition, and volume ratio. The use of optical signals to transmit sensing data constitutes a simple, cheap, and rapid strategy for processing information that can be readily multiplexed. Novel stimuli-responsive colloidal materials capable of reversibly modulating their light-reflecting, light-refracting, and light-emitting properties, such that they are capable of undergoing a trigger-induced switch between a translucent or opaque state, therefore promise to improve and extend the capabilities of current technologies.

Janus droplets are tunable liquid compound microlenses. [25] Depending on the curvature of the inner interface, collimated light that is transmitted through a droplet monolayer can be diverged or converged, where only one particular Janus morphology corresponds to a lens of infinite focal length. Thus, precise tuning of the curvature of the internal interface can vary the focal length and numerical aperture of Janus droplet lenses. Zarzar et al. exploited such instant changes in transmission for a straightforward design of a sensing platform for visualization and quantification of enzyme activity. [49].

Depending on the incident angle of illumination and the droplet morphology, incoming light rays can also be guided along the internal interface through total internal reflection increasing the light intensity inside droplets’ higher refractive index phase. [58] The latter results in an increased light intensity in proximity to the internal droplet interface, and droplet layers in defined geometries display a unique reflectivity analogous to corner cube reflectors. [59] When Janus droplets in uniform internal morphologies are illuminated with collimated white light from defined angles, optical interference occurring between light rays of different wavelengths traveling by different paths of total internal reflection result in the generation of iridescent structural color profiles through optical interference. [60] Trajectories of light undergoing refraction at the external droplet interface followed by retroreflection at the concave internal droplet interface can further result in an angle-dependent structural color signal output. [61] Consequently, non-dyed Janus droplets with concave internal interfaces generate colors that can be varied solely by modulating the internal droplet morphologies. While Janus droplets may be brightly iridescent, double emulsions do not reflect color, an effect that can be used to report chemically induced alterations in droplet morphology. [62].

Alongside variations in the Janus emulsion transmission and reflection properties, Janus droplets have also been demonstrated as powerful stimuli-responsive fluorescent probes. The tunable refractive index contrast between the constituent phases enables the attenuation or enhancement of embedded fluorescent dye intensity that selectively partition into one or both droplet phases. [26] When a fluorescent dye is localized into the higher refractive index droplet phase, total internal reflection confines the emission resulting in a strongly anisotropic angular fluorescence intensity distribution. [26] Theoretical ray-tracing calculations supported and explained the strong dependency of the angular emission profiles on the droplets’ interfacial curvatures and refractive index contrasts. Exploiting these fluorescence characteristics of dyed Janus emulsions, sensitive, dosimetric, and multiplexed read-out platforms capable of reporting marginal changes in the internal droplet shape could be realized. [26, 56, 63].

Alternative fluorescent droplet morphology read-out platforms build upon a compartmentalization of two complementary dyes or pigments into the opposing phases of Janus emulsions. These paradigms ratiometrically detect multiple emissions modulated by an inner filter effect that depends on the internal droplet morphology. [59, 64] In contrast to embedding fluorescent dyes into one or both of the bulk phases of Janus emulsions, interfacial functionalization and localization of fluorescent dyes selectively at one of the interfaces of Janus emulsions provides an alternative fluorescent read-out paradigm. [50, 65] These transduction mechanisms exploit the ability of Janus emulsions to present and hide liquid interfaces selectively. Lin et al. demonstrated control over a hydrogen-bonding mediated fluorescence quenching of surfactants based on a meta-amino substituted green fluorescence protein chromophore. [50].

**Fig. 5:**
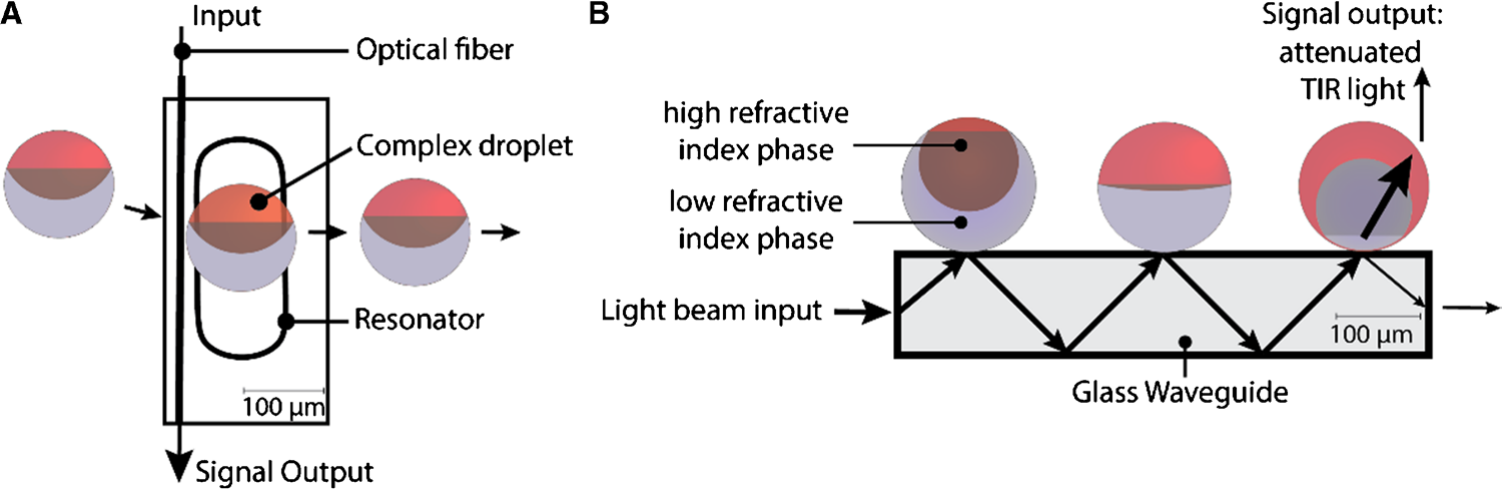
Modular Janus droplet sensing layers for refractive index–based sensing platforms based on (**a**) microcavity resonators and (**b**) optical waveguides. Figure sketches adapted from references [47, 66]

Researchers have further pioneered alternative purely refractive index–based signal transduction paradigms that are not based on colorimetric or fluorescent detection of droplet morphology. [47, 66] Morphology-dependent interactions of the emulsion droplets with an evanescent wave emerging from optical waveguides or microcavity resonators expand the capabilities of modular Janus droplet sensing layers to more complex, e.g., light-absorbing analyte environments, such as blood or tainted food samples (Fig. 5). Variations in droplet morphology create significant changes in the refractive index in proximity to the optical resonator, resulting in frustrated or attenuated total internal reflection of light, which outlines the versatility of Janus droplet–based modular sensing layers for liquid-phase, real-time, and continuous-flow detection of chemicals or biomolecules.

## Janus emulsion biosensors

**Fig. 6:**
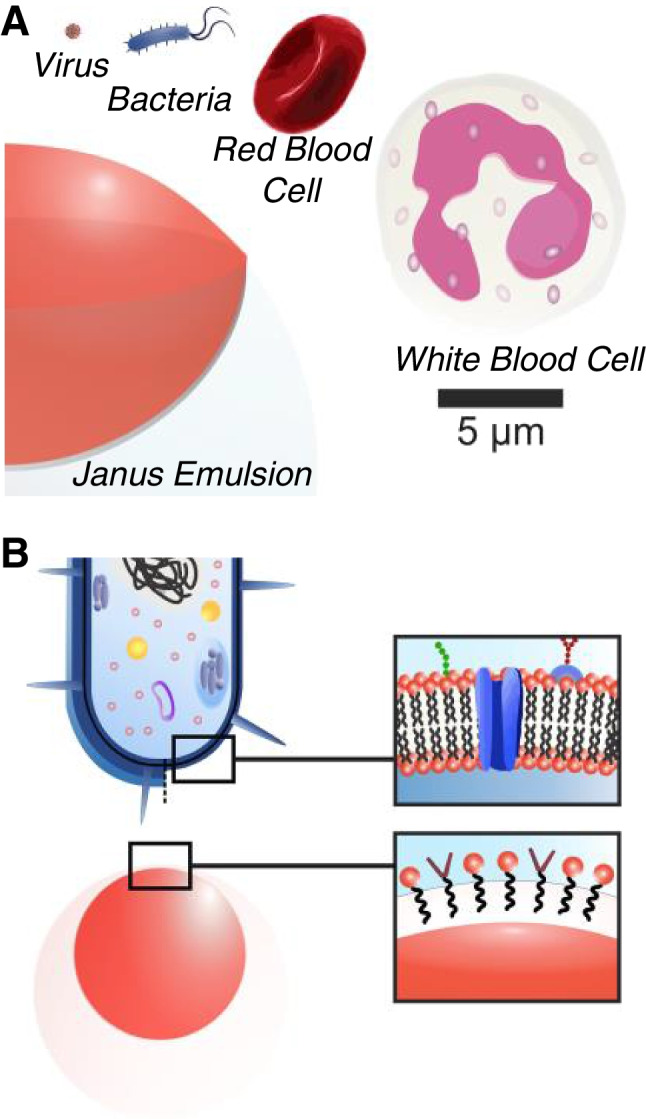
Similarities and differences in size and surface chemistry of Janus emulsions compared to biological entities. **A** Size comparison between Janus emulsion systems described in this article and biologicals. **B** Illustrative comparison of the surface chemistries of bacterial cell membranes versus surfactant-stabilized biphasic complex emulsions

The mechanical and interfacial properties of Janus emulsions exhibit similarity to living cells in that they present dynamic and reactive surfaces, multiple liquid compartments, and can chemically interact with their environment. Consequently, Janus emulsions offer an extraordinary untapped potential for real-time monitoring of interactions with biological entities and the development of new and improved biosensing platforms. In nature, binding and recognition phenomena on cell surfaces are often regulated by a series of cell-surface receptors, which provide their fundamental capability to transduce and convey chemical information autonomously and with high fidelity. These constitute essential processes by which the body maintains control over complex biological functions. [67, 68] A large number of such physiological and pathogenic events are directed and mediated through complex networks of protein–protein and protein-carbohydrate interactions. Thus, expanding the above-described Janus emulsion chemical sensor paradigms towards a dynamic and reversible immobilization of cellular recognition motifs at the droplet interfaces enables to biomimetically emulate such complex recognition and signal transduction processes within a novel artificial, cell-sized, and synthetically minimal fluid material platform that exhibits cell-like mechanical and interfacial properties (Fig. 6).

The implications of Janus emulsion–based sensor paradigms are profound as they offer a disruptive new biosensing strategy for rapidly and inexpensively identifying small amounts of pathogenic organisms. Sensory devices that utilize the Janus emulsion–based detection schemes can be readily miniaturized, and the emulsions are easily and cheaply prepared in any environment. In addition, the fundamental components of any biosensor, a recognition, transduction, and response unit are combined within one material system. A targeted combination of the unique chemical, morphological, and optical properties of Janus emulsions provides a multitude of potential signaling pathways for the precise detection and identification of pathogenic organisms and signal amplification in sensing devices.

Thus far, researchers have focused on two different signal transduction pathways underpinned by an immobilization of biochemically active surfactants at selective droplet interfaces. In these sensing paradigms (Table 1), molecular and cellular recognition events either cause morphological droplet responses via changing the balance of interfacial tensions (morphological assay) or multivalent binding interactions evoke droplets to tilt out of their gravitational alignment, whereas their morphology remains unchanged (agglutination assay).

**Table 1:** Overview of reported Janus emulsion–based optical biosensing platforms

Entry	Target pathogen	Approximate limit of detection	Time-to-results	Biochemical recognition strategy	Optical read-out	Read-out device	Ref
Agglutination assays							
1	*Escherichia coli*	10^4^ CFU mL^−1^	2 h	Carbohydrate-lectin binding	Scattering	Smartphone (via QR-code)	[48]
2	*ConA (as E. coli mimic)*	-	-	Carbohydrate-lectin binding	Refractive index change	Optical waveguide	[47]
3	Salmonella enterica (HKST*)	10^5^ cells mL^−1^	-	Antibody-antigen binding	Scattering	Smartphone (via QR-code)	[53]
4	*Zika virus (Zika NS1 protein)*	100 nM	12 h	Protein–protein interactions	Backscattering/corner cube reflectivity	Fiber-optic spectrometer	[59]
5	*Zika virus (Zika NS1 protein)*	100 nM	12 h	Protein–protein interactions	Inner filter effect	Fluorescence spectrometer	[59]
6	Listeria monocytogenes (HKLM*)	10^2^ cells mL^−1^	2 h	Antibody-antigen binding	Inner filter effect	Fluorescence spectrometer	[64]
7	*Anti-SARS-CoV-2 spike antibody*	0.2 µg mL^−1^	2 h	Antibody-antigen binding	Inner filter effect	Fiber-optic spectrometer	[69]
Morphological assays							
8	*Not tested*	-	-	Enzymatic surfactant cleavage (amylase, lipase, sulfatase)	Transmission	Fiber-optic spectrometer	[49]
9	*Salmonella enterica*	10^3^ CFU mL^−1^	2 h	Carbohydrate-lectin binding	Emission intensity (L-curve)	Fiber-optic spectrometer	[26]
10	*Salmonella enterica*	10^2^ CFU mL^−1^	2 h	Antibody-antigen binding	Emission intensity (L-curve)	Fiber-optic spectrometer	[26]
11	*Not tested*	-	-	Enzymatic surfactant cleavage (trypsin)	Emission intensity	Fluorescence spectrometer	[50]
12	Not tested	-	-	Enzymatic polymer cleavage (dextranase)	-	Side-view microscopy	[38]
13	Not tested	-	-	Enzymatic surfactant release (amylase)	Iridescent color change	Smartphone camera	[62]
14	Salmonella enterica (HKST*)	10^3^–10^4^ cells mL^−1^	3 h	Antibody-antigen binding	Color change/liquid crystal organization	Fiber-optic spectrometer	[70]
15	Not tested	-	-	Surfactant oxidation: oxidase	Emission intensity (L-curve)	Fiber-optic spectrometer	[63]

*Heat-killed bacteria cells

In Janus emulsion morphological assays, the detection strategy relies on reversible reactions between biological targets with droplet interface-immobilized selectors, which triggers changes in the balance of interfacial tensions and thus actuate dynamic reconfigurations in droplet morphology. Such changes in droplet morphology can be readily detected by an alteration of the optical properties of Janus emulsions exploiting the unique refractive, reflective, or light-emissive properties. For instance, leveraging an orthogonal carbohydrate binding of sugar-binding lectins at pathogen cell-surface appentages can be used to alter the droplet interfacial tensions thereby inducing large and predictable changes in the Janus emulsion morphology. [26] For this purpose, small molecule and polymeric boronic acid surfactants were synthesized and immobilized at selective droplet interfaces, which undergo dynamic covalent associations with carbohydrates. Competitive binding interactions with bacterial cells’ lectin protein receptors transduced into a change in droplet configuration, which could be detected via changes in Janus droplet fluorescence intensity. Specifically, the phenomenon of total internal reflection of light emitted from the higher refractive index droplet phase creates a morphology-dependent anisotropic emission signature that allows the detection of minuscule variations in the droplet morphology. The approach could be extended towards an oriented side-selective immobilization of antibodies to demonstrate rapid and sensitive detection of Salmonella bacteria with low detection thresholds of < 100 cells/mL. Similar transduction mechanisms were used in sensing platforms targeting other analytes. [56, 63, 70, 71].

In turn, Janus droplet agglutination assays build upon a chemically induced tilting of Janus droplets out of their gravitational alignment that can be evoked by multivalent binding interactions with an analyte. [48] In these assays, the internal droplet morphology remains unaffected. Agglutination schemes require a side-selective surface-immobilization of active components that bind multivalently to a host analyte within the continuous aqueous phase. In analogy to the lectin binding paradigms described above, a covalent attachment of carbohydrates to the droplet interface can induce a lectin-triggered agglutination of droplets. [72] Janus droplets orient with gravity, placing the denser fluorocarbon phase at the bottom. However, upon addition of lectins, multivalent binding of the surface-immobilized carbohydrate units results in a tilting of droplet networks. [47, 59] Agglutination can be readily observed through an increased scattering of droplet layers, which can be quantified via image analysis, e.g., using a smartphone camera. [48].

## Limitations and prospects of Janus droplet transducers

Research in the field of biosensor development is very active, and there are many reports on the application of rapid sensing techniques for the detection of various bacteria and viruses, focusing on both medical diagnostics and the monitoring of pathogens in food, water, and the environment. [73] There are a wide range of highly developed detection methods available, which include ELISA, PCR and other immunoassays, mass-based identification techniques, aptamer-based assays, as well as a variety of elegantly designed nanomaterial-based biosensors that can detect pathogens rapidly and with very low detection thresholds. [74–78] In practice, the feasibility of any method for the identification of pathogens in a particular application setting is determined by a combination of trade-off factors, such as the specificity and sensitivity of the detection method, its speed of detection, the availability of the necessary instrumentation, and, in particular also financial considerations. [79] The latter is of particular importance in the context of monitoring food safety. For instance, while PCR techniques are a standard technique in medical diagnostics for identifying many different bacteria and viruses with extremely low detection thresholds, extracting, amplifying, and identifying DNA from water or food is more difficult, especially at low pathogen concentrations. Lower amounts of target nucleic acids require laborious extraction and amplification and this intense investment of time and resources is incompatible with the needs of rapid, on-site food safety monitoring. In addition, price pressure on end products demands a simple as cost-effective screening technique. As a consequence, the majority of food tests to date are performed using culture-based means of identification despite the associated time delay in obtaining a positive result. [21, 80].

Consideration of this need for simplicity highlights that modular sensory layers based on Janus emulsions, although still in their infancy, hold promise to address an unmet need for the development of rapid screening platforms for monitoring food safety. Modular Janus emulsion transduction platforms can be easily and inexpensively prepared from low-cost starting materials in almost any environment. In addition, the unique morphological-optical coupling inside Janus droplets allows facile optical transduction of chemical information in situ, alleviating the need for complicated read-out instrumentation, which lays the foundation for their implementation in simple, low-cost, and high-speed on-site monitoring devices.

However, despite their promising potential, there are major challenges that Janus droplet modular sensor platforms need to overcome before discussing their full potential and suitability for practical applications. First and foremost, the stability of droplets and their sensitivity towards detecting pathogens in complex food matrices need to be explored. The unique dynamic behavior of droplets is underpinned by their kinetic stabilization, which, however, conditions their susceptibility to mechanical rupture as well as Ostwald ripening, flocculation or coalescence. Thus, while Janus drops can be produced cheaply and easily, production scalability, distribution and long-term storage may be compromised. With respect to detection efficiency, a continued merger of dynamic Janus droplet interface sensitization with known recognition motifs from supramolecular chemistry, microbiology, or catalysis promises to improve the specificity and sensitivity towards specific pathogens. Hitherto reported paradigms achieved moderate detection thresholds that, while not suitable for the requirements in medical diagnostics, already meet benchmarked target ranges and general sensitivity requirement for the detection of a number of bacterial strains from food samples. [81] However, the extent to which Janus droplet–based sensor devices will reach a level of sophistication to recognize single specific organisms within complex mixtures, to distinguish between live and dead bacteria cells, and to function in opaque and light-absorbing media remains to be explored. The latter poses a particular challenge for optical read-out paradigms. Optical transduction methods are characterized by their simplicity and ease of operation, but continuous monitoring of complex droplets in real-time and generating multiplexed signals in opaque and light-absorbing media is inherently more complicated. Advanced read-out platforms that extend beyond measuring droplet samples in a discrete batch-to-batch manner will be central to progressing this technology. [56].

Beyond necessary improvements in sensitivity, specificity, and practicability of sensing platforms to track singular events, a particular and unprecedented inherent feature of Janus droplet transducers lies in a more general strategy for tracking multiple independent (bio-)chemical interactions simultaneously, rather than tracking singular events. Instead of designing systems that fulfill a single targeted responsive function, this multifunctional system allows deciphering the structural and energetic basis to track multiple autonomous processes in parallel. [46] Underpinned by a variety of independent responsive modalities, Janus droplets impart the capability to act as adaptive local force sensors, portraying their immediate chemical environment. Regardless of the particular transduction mechanisms, which could include morphological and agglutinating events as well as chemotactic behaviors amongst others, [82] Janus droplets possess an intrinsic ability to respond independently to multiple chemical interactions at their interface, [56] which means that they could, in principle, also be able to process this information.

Consequently, perhaps the biggest strength and true power of the modular Janus droplet–based transduction layers lies in the dynamic, multicompartmentalized nature of the liquid material itself. Unlike many other transduction techniques, this liquid sensing platform enables signal transduction that goes beyond a simple translation of a chemical detection event into a physical readout. Janus emulsion droplets intrinsically represent a thermodynamically out-of-equilibrium material platform that continuously exchanges molecules with its environment. [83–85] Programmable regulation of their structural and functional changes to a wide variety of changes in their chemical environment could thus increase the complexity and specificity of these sensing systems and further allow to incorporate multiple gating and storage capacities. Multifunctional, adaptive droplet ensembles could controllably harness different energy gradients induced by multiple independent chemical binding events, thus paving the path towards the design of sensing platforms that release interpreted signals.

This vision of droplets exhibiting a decentralized chemical intelligence emulates design principles that are common in nature. Often, the key to the high specificity and sensitivity of regulated responses in nature lies in their ability to design interconnected adaptable molecular and supramolecular networks that recognize, propagate, and transduce chemical and biochemical information with high fidelity. Current Janus droplet sensor platforms have obviously not reached this level of complexity. Introducing multiple responsive components into a single system, however, will yield transducers that release informed responses balancing positive and negative feedback loops. In the context of sensing, the challenge then lies in achieving programmed control over this chemical autonomy of the systems and controlling the superpositions of singularities to create an informed sensor data output signal.

## Outlook

Currently, we can conveniently generate large quantities of small Janus droplets in highly uniform internal morphologies, control their assembly, reversibly actuate a tilting or chemotactic motility of droplet ensembles, trigger their coalescence, and under certain conditions, manipulate droplet geometry and the reversible uptake and release of chemical contents while monitoring all these processes in real-time. Advances in the compositional flexibility and complexity of the emulsion droplets may pave the path towards the generation of more chemo-intelligent micro-colloids that display multiple responsive units, can communicate with each other, or that display active, adaptive, self-regulatory, and autonomous capabilities, e.g., in the context of achieving spatial and temporal control over chemical reaction cascades. This is analogous to nature’s liquid colloidal systems, where the highest substrate specificities are often not achieved through single recognition events but via a sophisticated positional assembly and spatial separation of biologically or catalytically active reaction centers. Multiple triggering events then transduce into an actuation of a specific reaction cascade, e.g., leading to an immunological response. Similarly, sophisticated artificial Janus droplet–based intelligent multicomponent systems could regulate and modulate chemical reactivity and provide the highest degrees of sensitivity and specificity to liquid-based sensing platforms.

Due to their dynamic liquid nature and intrinsic structural responsivity to marginal variations in the force balance of interfacial tensions, Janus emulsions possess an acute sense of their chemical environment. Building on the foundation of both morphological and agglutination assays, advances in the sophistication by which Janus emulsions can be functionalized, stabilized, and organized will certainly be central to evolving droplet-based biosensor technologies. The ability to prepare stimuli-responsive Janus droplets easily and cheaply in any environment, combined with read-out techniques that do not require sophisticated and costly laboratory settings, facilitate in situ monitoring of chemical reactions at the interface and thus provide particular benefits for the development of rapid, on-site detection platforms with increased throughput, distribution, and controllability of assays. For practical considerations, which include environmental sampling, such platforms could involve disposable swab-based test vials with the necessary components distributed in a solution complemented by a locally installed imaging platform. As a result, their miniaturization and distribution could be accomplished through established shipment pathways. Despite this promising potential, the application of Janus emulsion droplets in large-scale single-use swab-based screening devices, that function similarly fast and sophisticated as, e.g., drug screening platforms at airports or antigen test strips, will require a keen understanding of the underlying physico-chemical, morphological, optical, thermodynamic, and dynamic properties of the multicompartment liquid colloids in different environments.

The modularity by which the individual interfaces and phase boundaries can be functionalized will further stimulate the rapid development of alternative transduction mechanisms. The unique chemical-morphological-optical coupling inside Janus droplets offers rich opportunities to develop optical read-out concepts. The unique refractive optical properties of the reactive Janus droplets are highlighted by the gravitational alignment of the individual droplet phases and the adjustable refractive index contrast of the dispersed phase liquids. As a result, Janus droplets can manipulate the transmitted light pathway and exhibit intriguing angular anisotropic refraction, reflection, and emission features. Variations in the collected or emitted light intensities of Janus droplet layers can be easily monitored visually or quantitatively using simple fiber-optic setups, e.g., using the light meter of a smartphone camera. Stimuli-responsive colloidal materials that can undergo a trigger-induced switch between a translucent and opaque state, or to reversibly modulate their reflective, refractive, or emissive properties promise to improve and expand the capabilities of current technologies. As such, modular sensing layers based on Janus droplets hold promise to expand the scope and decrease the signal-to-noise ratio in several established refractive index–based sensing technologies, including total internal reflection fluorescence microscopes, dual polarization interferometers, plasmonic resonators, resonant microcavities, or colorimetric iridescent structural color sensing arrays.

Today, most Janus emulsion–based sensor paradigms are underpinned by sensitization of droplet interfaces with stimuli-responsive surfactants that are sensitive towards the detection of particular analytes. The design of multi-responsive droplet sensors provides the potential to detect multiple analytes simultaneously and thus to sense complex environments. Modular optically active Janus droplet sensor layers offer facile implementation into sensor arrays, and an intrinsic advantage of optical read-out techniques is that they can be easily multiplexed. Building on the responsive properties that translate external stimuli into a binary on–off state, future efforts will further be directed towards demonstrating the ability of Janus droplets to self-regulate and adapt to multiple triggers in their chemical environment. Biphasic droplets in which more than one stimuli triggers microscale morphological transitions and, in turn, macroscale optical outputs signals could then be used to expand from simple YES or NOT read-outs to building AND/OR logic gate sensor circuits.

This is an exciting perspective because Janus droplets can display multiple independent responsive modalities. Next to morphological transitions, droplets can interact with their immediate chemical environment also by reversibly attaching to surfaces, multivalently binding to each other, or exchanging mass non-reciprocally, e.g., mediated by osmotic pressure differences. [46, 85, 86] In addition, Janus droplets are capable to undergo trigger-induced chemotactic motion that is mediated either by micellar solubilization of the dispersed phases or chemically evoked interfacial tension gradients within the surrounding continuous phase. [82, 87] The speed and directionality of the overall droplet motion can be reversibly and controllably altered depending on the internal morphology of the droplets. [82] This lays the foundation for the design of droplets that display programmable motion towards or away certain chemoattractants or -repellents, yielding novel types of motile sensors capable to hunt after their targets.

Targeting two or more of these separate responsive modalities within a single droplet system will yield adaptive droplet ensembles. The concept of adaptivity requires responsive materials capable to independently and autonomously regulate their responses to different input signals, such as molecular recognition–induced chemical events. [88] In such systems, multiple individual or combinations of independent chemical equilibrium–driven transformations are then translated into a specific response. Droplets capable to dynamically regulate their response and adapt to their environment give rise to speculate about the design of artificial, autonomously operating, chemo-intelligent life-like soft colloids that can perform collective functions and thus exhibit strong implications for future sensor technologies. In such highly dynamic systems, simultaneous tracking of multiple responsive modalities for successful identification of specific analytes within complex environments poses a major challenge, and sophisticated analysis of the collected sensor data that goes beyond simple decision trees will be required for successful differentiation.

Clearly, these approaches will require multidisciplinary collaborative efforts from physicists, chemists, biologists, and engineers, and the intention of this article is to provide a resource and motivate future research developments in advancing concepts and improving biochemical detection paradigms, signal transduction, amplification, and readout technologies. The vision is a facile generation of artificial chemo-intelligent soft colloids that can ultimately rival the complexity and regulatory capabilities of natural systems, which will enable new sensory methods that may surpass the performance of other biochemical sensor technologies in their selectivity, sensitivity, and stability, while maintaining the simplicity and cost-effectiveness of Janus droplet–based modular sensory layers.

## References

[1] World Health Organization. WHO estimates of the global burden of foodborne diseases: foodborne disease burden epidemiology reference group 2007–2015: World Health Organization; 2015.

[2] Connelly JT, Baeumner AJ (2012). Biosensors for the detection of waterborne pathogens. Anal Bioanal Chem.

[3] Buzby JC, Roberts T, Lin CTJ, MacDonald JM (1996). Bacterial foodborne disease: medical costs and productivity losses.

[4] Velusamy V, Arshak K, Korostynska O, Oliwa K, Adley C (2010). An overview of foodborne pathogen detection: in the perspective of biosensors. Biotech Adv.

[5] International Organization for Standardization (2006). Microbiology of food and animal feeding stuffs — horizontal method for the enumeration of coliforms — colony-count technique. ISO.

[6] Stephan R, Schumacher S, Zychowska MA (2003). The VIT® technology for rapid detection of Listeria monocytogenes and other Listeria spp. Int J Food Microbio.

[7] Scharff RL (2020). Food attribution and economic cost estimates for meat- and poultry-related illnesses. J Food Protection.

[8] Furst AL, Francis MB (2019). Impedance-based detection of bacteria. Chem Rev.

[9] McEachern F, Harvey E, Merle G (2020). Emerging technologies for the electrochemical detection of bacteria. Biotechn J.

[10] Anany H, Brovko L, El Dougdoug NK, Sohar J, Fenn H, Alasiri N (2018). Print to detect: a rapid and ultrasensitive phage-based dipstick assay for foodborne pathogens. Anal Bioanal Chem.

[11] Mi F, Hu C, Wang Y, Wang L, Peng F, Geng P (2022). Recent advancements in microfluidic chip biosensor detection of foodborne pathogenic bacteria: a review. Anal Bioanal Chem.

[12] Zhang J, Wang Y, Lu X (2021). Molecular imprinting technology for sensing foodborne pathogenic bacteria. Anal Bioanal Chem.

[13] Asiello PJ, Baeumner AJ (2011). Miniaturized isothermal nucleic acid amplification, a review. Lab Chip.

[14] Hameed S, Xie L, Ying Y (2018). Conventional and emerging detection techniques for pathogenic bacteria in food science: a review. Trends Food Sci Technol.

[15] Fenzl C, Hirsch T, Baeumner AJ (2016). Nanomaterials as versatile tools for signal amplification in (bio)analytical applications. Trends Anal Chem.

[16] Wang C, Gao X, Wang S, Liu Y (2020). A smartphone-integrated paper sensing system for fluorescent and colorimetric dual-channel detection of foodborne pathogenic bacteria. Anal Bioanal Chem.

[17] Creran B, Li X, Duncan B, Kim CS, Moyano DF, Rotello VM (2014). Detection of bacteria using inkjet-printed enzymatic test strips. ACS Appl Mater Interfaces.

[18] Vinayaka AC, Thakur MS (2010). Focus on quantum dots as potential fluorescent probes for monitoring food toxicants and foodborne pathogens. Anal Bioanal Chem.

[19] Sivakumar S, Wark KL, Gupta JK, Abbott NL, Caruso F (2009). Liquid crystal emulsions as the basis of biological sensors for the optical detection of bacteria and viruses. Adv Funct Mater.

[20] Locke A, Fitzgerald S, Mahadevan-Jansen A (2020). Advances in optical detection of human-associated pathogenic bacteria. Molecules.

[21] Batt CA (2007). Food Pathogen Detection. Science.

[22] Lohse D, Zhang X (2020). Physicochemical hydrodynamics of droplets out of equilibrium. Nat Rev Phys.

[23] Choi CH, Kim J, Nam JO, Kang SM, Jeong SG, Lee CS (2014). Microfluidic design of complex emulsions. ChemPhysChem.

[24] Balaj RV, Zarzar LD (2020). Reconfigurable complex emulsions: design, properties, and applications. Chem Phys Rev.

[25] Nagelberg S, Zarzar LD, Nicolas N, Subramanian K, Kalow JA, Sresht V (2017). Reconfigurable and responsive droplet-based compound micro-lenses. Nat Commun.

[26] Zeininger L, Nagelberg S, Harvey KS, Savagatrup S, Herbert MB, Yoshinaga K (2019). Rapid detection of Salmonella enterica via directional emission from carbohydrate-functionalized dynamic double emulsions. ACS Centr Sci.

[27] Torza S, Mason S (1969). Coalescence of two immiscible liquid drops. Science.

[28] Hasinovic H, Friberg SE (2011). One-step inversion process to a Janus emulsion with two mutually insoluble oils. Langmuir.

[29] Mabille C, Schmitt V, Gorria P, Leal Calderon F, Faye V, Deminière B (2000). Rheological and shearing conditions for the preparation of monodisperse emulsions. Langmuir.

[30] Hasinovic H, Friberg SE, Rong G (2011). A one-step process to a Janus emulsion. J Coll Interf Sci.

[31] Fryd MM, Mason TG (2013). Cerberus nanoemulsions produced by multidroplet flow-induced fusion. Langmuir.

[32] Utada AS, Lorenceau E, Link DR, Kaplan PD, Stone HA, Weitz DA (2005). Monodisperse double emulsions generated from a microcapillary device. Science.

[33] Shah RK, Shum HC, Rowat AC, Lee D, Agresti JJ, Utada AS (2008). Designer emulsions using microfluidics. Mater Today.

[34] Zarzar LD, Sresht V, Sletten EM, Kalow JA, Blankschtein D, Swager TM (2015). Dynamically reconfigurable complex emulsions via tunable interfacial tensions. Nature.

[35] Frank BD, Perovic M, Djalali S, Antonietti M, Oschatz M, Zeininger L (2021). Synthesis of polymer Janus particles with tunable wettability profiles as potent solid surfactants to promote gas delivery in aqueous reaction media. ACS Appl Mater Interfaces.

[36] Frank BD, Antonietti M, Zeininger L (2021). Structurally anisotropic Janus particles with tunable amphiphilicity via polymerization of dynamic complex emulsions. Macromol.

[37] Ku KH, Li J, Yoshinaga K, Swager TM (2019). Dynamically reconfigurable, multifunctional emulsions with controllable structure and movement. Adv Mater.

[38] Pavlovic M, Antonietti M, Schmidt BVKJ, Zeininger L (2020). Responsive Janus and Cerberus emulsions via temperature-induced phase separation in aqueous polymer mixtures. J Coll Interf Sci.

[39] Guzowski J, Korczyk PM, Jakiela S, Garstecki P (2012). The structure and stability of multiple micro-droplets. Soft Matter.

[40] Li T, Schofield AB, Chen K, Thijssen JH, Dobnikar J, Clegg P (2019). Particle-stabilized Janus emulsions that exhibit pH-tunable stability. Chem Commun.

[41] Concellón A, Zentner CA, Swager TM (2019). Dynamic complex liquid crystal emulsions. J Amer Chem Soc.

[42] Wang X, Zhou Y, Kim Y-K, Tsuei M, Yang Y, de Pablo JJ (2019). Thermally reconfigurable Janus droplets with nematic liquid crystalline and isotropic perfluorocarbon oil compartments. Soft Matter.

[43] Kovach I, Friberg SE, Koetz J (2017). A “perfect Janus emulsion”: thermodynamic factors. J Dispersion Sci Technol.

[44] Djalali S, Frank BD, Zeininger L (2020). Responsive drop method: quantitative in situ determination of surfactant effectiveness using reconfigurable Janus emulsions. Soft Matter.

[45] Zhao J, Pan Z, Snyder D, Stone HA, Emrick T (2021). Chemically triggered coalescence and reactivity of droplet fibers. J Amer Chem Soc.

[46] Djalali S, Simón Marqués P, Frank BD, Zeininger L (2022). Crown ether-functionalized complex emulsions as an artificial adaptive material platform. Adv Funct Mater.

[47] Zeininger L, Weyandt E, Savagatrup S, Harvey KS, Zhang Q, Zhao Y (2019). Waveguide-based chemo- and biosensors: complex emulsions for the detection of caffeine and proteins. Lab Chip.

[48] Zhang Q, Savagatrup S, Kaplonek P, Seeberger PH, Swager TM (2017). Janus emulsions for the detection of bacteria. ACS Centr Sci.

[49] Zarzar LD, Kalow JA, He X, Walish JJ, Swager TM (2017). Optical visualization and quantification of enzyme activity using dynamic droplet lenses. Pro Nat Acad Sci.

[50] Lin C-J, Zeininger L, Savagatrup S, Swager TM (2019). Morphology-dependent luminescence in complex liquid colloids. J Amer Chem Soc.

[51] Fernandez A, Zentner CA, Shivrayan M, Samson E, Savagatrup S, Zhuang J (2020). Programmable emulsions via nucleophile-induced covalent surfactant modifications. Chem Mater.

[52] James TD, Sandanayake K, Shinkai S (1996). Saccharide sensing with molecular receptors based on boronic acid. Angew Chem Int Ed.

[53] Zentner CA, Anson F, Thayumanavan S, Swager TM (2019). Dynamic imine chemistry at complex double emulsion interfaces. J Amer Chem Soc.

[54] Zentner CA, Concellón A, Swager TM (2020). Controlled movement of complex double emulsions via interfacially confined magnetic nanoparticles. ACS Centr Sci.

[55] Pavlovic M, Ramiya Ramesh Babu HK, Djalali S, Vraneš M, Radonić V, Zeininger L (2021). Facile monitoring of water hardness levels using responsive complex emulsions. Anal Chem..

[56] Barua B, Durkin TJ, Beeley IM, Gadh A, Savagatrup S (2023). Multiplexed and continuous microfluidic sensors using dynamic complex droplets. Soft Matter.

[57] Pavlovic M, Ramiya Ramesh Babu HK, Djalali S, Pavlovic Z, Vraneš M, Zeininger L (2023). Dynamic in situ monitoring of the salt counter-ion effect on surfactant effectiveness using reconfigurable Janus emulsions. Langmuir..

[58] Simón Marqués P, Frank BD, Savateev A, Zeininger L (2021). Janus emulsion solar concentrators as photocatalytic droplet microreactors. Adv Opt Mater.

[59] Zhang Q, Zeininger L, Sung K-J, Miller EA, Yoshinaga K, Sikes HD (2019). Emulsion agglutination assay for the detection of protein–protein interactions: an optical sensor for Zika virus. ACS Sensors.

[60] Goodling AE, Nagelberg S, Kaehr B, Meredith CH, Cheon SI, Saunders AP (2019). Colouration by total internal reflection and interference at microscale concave interfaces. Nature.

[61] Yang Y, Kim JB, Nam SK, Zhang M, Xu J, Zhu J (2023). Nanostructure-free crescent-shaped microparticles as full-color reflective pigments. Nat Commun.

[62] Saunders AP, Zarzar LD. Structural coloration from total internal reflection at microscale concave surfaces and use for sensing in complex droplets. Advanced Fabrication Technologies for Micro/Nano Optics and Photonics. 2020;11292. 10.1117/12.2545151

[63] Fong D, Swager TM (2021). Trace detection of hydrogen peroxide via dynamic double emulsions. J Amer Chem Soc.

[64] Li J, Savagatrup S, Nelson Z, Yoshinaga K, Swager TM (2020). Fluorescent Janus emulsions for biosensing of Listeria monocytogenes. Proc Nat Acad Sci.

[65] Marqués PS, Krajewska M, Frank BD, Prochaska K, Zeininger L. Morphology-dependent aggregation-induced emission of Janus emulsion surfactants. Chem Eur J. 2023;29(18):e202203790. 10.1002/chem.202203790 10.1002/chem.202203790 36661211

[66] Savagatrup S, Ma D, Zhong H, Harvey KS, Kimerling LC, Agarwal AM (2020). Dynamic complex emulsions as amplifiers for on-chip photonic cavity-enhanced resonators. ACS Sensors.

[67] Qi Z, Bharate P, Lai C-H, Ziem B, Böttcher C, Schulz A (2015). Multivalency at interfaces: supramolecular carbohydrate-functionalized graphene derivatives for bacterial capture, release, and disinfection. Nano Lett.

[68] Lis H, Sharon N (1998). Lectins: carbohydrate-specific proteins that mediate cellular recognition. Chem Rev.

[69] Li J, Concellón A, Yoshinaga K, Nelson Z, He Q, Swager TM (2021). Janus emulsion biosensors for anti-SARS-CoV-2 spike antibody. ACS Centr Sci.

[70] Concellón A, Fong D, Swager TM (2021). Complex liquid crystal emulsions for biosensing. J Amer Chem Soc.

[71] Trinh V, Malloy CS, Durkin TJ, Gadh A, Savagatrup S (2022). Detection of PFAS and fluorinated surfactants using differential behaviors at interfaces of complex droplets. ACS Sensors.

[72] Zhang Q, Scigliano A, Biver T, Pucci A, Swager TM (2018). Interfacial bioconjugation on emulsion droplet for biosensors. Bioorg Med Chem.

[73] Law JW-F, Ab Mutalib N-S, Chan K-G, Lee L-H (2015). Rapid methods for the detection of foodborne bacterial pathogens: principles, applications, advantages and limitations. Front Microbiol..

[74] Kabiraz MP, Majumdar PR, Mahmud MMC, Bhowmik S, Ali A (2023). Conventional and advanced detection techniques of foodborne pathogens: a comprehensive review. Heliyon.

[75] Aladhadh M (2023). A review of modern methods for the detection of foodborne pathogens. Microorganisms.

[76] Hofmann C, Duerkop A, Baeumner AJ (2019). Nanocontainers for analytical applications. Angew Chem Int Ed.

[77] Yang S, Rothman RE (2004). PCR-based diagnostics for infectious diseases: uses, limitations, and future applications in acute-care settings. Lancet Infect Dis.

[78] Zhou J, Battig MR, Wang Y (2010). Aptamer-based molecular recognition for biosensor development. Anal Bioanal Chem.

[79] Saravanan A, Kumar PS, Hemavathy RV, Jeevanantham S, Kamalesh R, Sneha S (2021). Methods of detection of food-borne pathogens: a review. Environ Chem Lett.

[80] Nugen S, Baeumner A (2008). Trends and opportunities in food pathogen detection. Anal Bioanal Chem.

[81] Commission E (2005). Commission Regulation (EC) No 2073/2005 of 15 November 2005 on microbiological criteria for foodstuffs. Off J Eur Union.

[82] Frank BD, Djalali S, Baryzewska AW, Giusto P, Seeberger PH, Zeininger L (2022). Reversible morphology-resolved chemotactic actuation and motion of Janus emulsion droplets. Nat Commun.

[83] Birrer S, Cheon SI, Zarzar LD (2022). We the droplets: a constitutional approach to active and self-propelled emulsions. Curr Opi Coll Interf Sci.

[84] Meredith CH, Moerman PG, Groenewold J, Chiu Y-J, Kegel WK, Van Blaaderen A (2020). Predator–prey interactions between droplets driven by non-reciprocal oil exchange. Nat Chem.

[85] Pavlovic M, Antonietti M, Zeininger L (2021). Cascade communication in disordered networks of enzyme-loaded microdroplets. Chem Commun.

[86] Nagelberg S, Totz JF, Mittasch M, Sresht V, Zeininger L, Swager TM (2021). Actuation of Janus emulsion droplets via optothermally induced Marangoni forces. Phys Rev Lett.

[87] Meredith CH, Castonguay AC, Chiu Y-J, Brooks AM, Moerman PG, Torab P (2022). Chemical design of self-propelled Janus droplets. Matter.

[88] Merindol R, Walther A (2017). Materials learning from life: concepts for active, adaptive and autonomous molecular systems. Chem Soc Rev.

